# A qualitative assessment of structural barriers to prenatal care and congenital syphilis prevention in Kern County, California

**DOI:** 10.1371/journal.pone.0249419

**Published:** 2021-04-01

**Authors:** Elaine Y. L. Chan, Carolyn Smullin, Stephanie Clavijo, Melissa Papp-Green, Eunhee Park, Marlene Nelson, Gloria Giarratano, Jennifer A. Wagman

**Affiliations:** 1 David Geffen School of Medicine at UCLA, Los Angeles, California, United States of America; 2 Department of Community Health Sciences, UCLA Fielding School of Public Health, Los Angeles, California, United States of America; 3 Charles R Drew University of Medicine and Science, Los Angeles, California, United States of America; 4 Los Angeles County Department of Public Health, Los Angeles, California, United States of America; 5 School of Social Sciences and Education, California State University-Bakersfield, Bakersfield, California, United States of America; 6 School of Nursing, Louisiana State University, Baton Rouge, Louisiana, United States of America; University of North Carolina at Chapel Hill, UNITED STATES

## Abstract

Congenital syphilis is the result of placental transmission from mother to fetus of *Treponema pallidum*. Although congenital syphilis is preventable through timely treatment, the rate of new infections in the United States (US) has increased each year since 2013, and is increasing at a noticeably greater pace in California (CA). Most research into congenital syphilis has focused on individual psychosocial and behavioral factors that contribute to maternal vulnerability for syphilis. The aim of this study was to evaluate structural barriers to prenatal care access and utilization and congenital syphilis prevention in Kern County, CA. Transcripts from 8 in-depth interviews with prenatal care providers and 5 focus group discussions with 42 pregnant and postpartum persons were examined using thematic analysis. Structural barriers experienced by pregnant and postpartum persons to prenatal care access and utilization included (1) burdens of poverty; (2) stigma around substance use in pregnancy; (3) citizenship status; (4) lack of healthcare coverage; (5) low sexual health literacy; and (6) gender inequality Structural barriers experienced by prenatal care providers in congenital syphilis prevention included (1) limited guidance on clinical management of syphilis in pregnancy; (2) decay in public health infrastructure; and (3) inadequate support for managing patients’ social comorbidities. The response to congenital syphilis prevention will require an examination of the complex context of social determinants of health in which persons diagnosed with syphilis live in.

## Introduction

Congenital syphilis is the result of placental transmission of the spirochete *Treponema pallidum* from an untreated or inadequately treated pregnant person to their infant. Congenital syphilis causes significant infant morbidity and mortality. The antepartum transmission of syphilis can result in spontaneous abortion and still birth, while live-born infants with congenital syphilis can develop musculoskeletal defects, sensorineural deafness and facial abnormalities [1].

The rate of congenital syphilis in the United States (US) has increased each year since 2013 [2]. In 2018, the national congenital syphilis case rate was 33.1 cases per 100,000 live births–a 39.7% increase relative to 2017 and a 185.3% increase relative to 2014. The majority of congenital syphilis cases in the US are concentrated in California (CA), Texas, Louisiana, Arizona and Nevada. In 2018, 329 cases of congenital syphilis were reported in CA—a 900% increase from the 33 total cases reported in California in 2012. These cases were distributed across Los Angeles (LA) (n = 64), Kern (n = 56), Fresno (n = 37), San Bernardino (n = 31), and San Joaquin (n = 26) counties [2, 3].

The rise in congenital syphilis incidence is part of a larger trend of increased syphilis infections [4]. The Centers for Disease Control and Prevention (CDC) recommends screening of all pregnant persons for syphilis at the first prenatal care visit, with additional screening during the third trimester and at delivery in areas or populations where the risk of syphilis is high [5]. While most states require first trimester testing, fewer states require testing later in pregnancy or at delivery [6]. Congenital syphilis cases are associated with late initiation and lack of prenatal care, and case rates are disproportionately high in the most socially vulnerable communities; social vulnerability and deprivation in mothers is often associated with an increase risk of inadequate prenatal care [6]. Numerous structural factors are related to lack of timely and adequate prenatal care, including lack of insurance, cost of co- pays, lack of transportation or child care, unawareness of the pregnancy, and mental health and substance use issues [4]. Despite an increasing recognition of the structural determinants of health–those determinants driven by political, social and economic forces which produce and maintain poor health outcomes–most research into congenital syphilis has focused on the individual psychosocial and behavioral factors that contribute to maternal vulnerability for syphilis. Very few published studies have attempted to examine how structural determinants of health contribute to congenital syphilis vulnerability [7–10].

We used qualitative methods to examine interviews with pregnant and postpartum persons and prenatal care providers in Kern County, CA to identify common themes regarding structural barriers that potentially contribute to lack of and untimely prenatal care access and utilization and inadequate congenital syphilis prevention.

## Materials and methods

### Participant eligibility and recruitment

This study was conducted as a qualitative investigation to assess factors that influence decision-making and behavior surrounding prenatal care and syphilis management among prenatal care providers and pregnant and postpartum persons in Kern County, CA. Kern County was selected because despite representing only 2.3% of CA state’s entire 2018 population, 17% of all congenital syphilis cases in CA were reported in Kern County [11]. A parallel study was conducted in East Baton Rouge, Louisiana and these findings have been published separately [12].

A relationship was established with all study partners and study participants prior to commencement. Participants were informed of the study aim: to learn about current prenatal care practices and beliefs in Kern County, a region of the US with high prevalence of congenital syphilis. Participants were informed of the study purpose: (1) to understand what interventions/programs could be developed and implemented to help pregnant women protect their own health and the health of their babies; and (2) to develop guidelines for prenatal care providers to effectively communicate with their patients at highest risk for syphilis infection and transmission so as to offer the best prevention and treatment services. Finally, participants were informed of the study goals: (1) to learn what women in communities with high prevalence of sexually transmitted infections do before pregnancy and during pregnancy to keep their babies healthy; and (2) to learn the professional practices of antenatal care providers, related to STI prevention and treatment, and where providers access information about STI prevention. No characteristics about the interviewer were reported.

All recruitment and data collection activities in the Kern County study setting were conducted by two bilingual (English-Spanish) masters level researchers from California State University, Bakersfield (hereafter referred to as field researchers) and the Principal Investigator (PI) of the study who was a faculty member at the University of California, San Diego School of Medicine (UCSD-SOM). Pregnant and postpartum persons were primarily recruited from a residential treatment program, a transitional sober living community, a domestic violence resource center, and from the general community of Spanish speaking individuals through use of flyers and posters in lobbies of respective recruitment sites. Some participants were recruited through snowball sampling, a nonrandom sampling technique whereby initial participants helped identify additional study participants. While this technique does not provide an equal opportunity for inclusion, we tried to avoid some of the biases inherent to snowball sampling by ensuring all participants (including both those purposefully selected by the research team, as well as those identified by existing participants) met a set of eligibility criteria. Eligibility criteria for pregnant and postpartum participants included: (1) 18 years and older; (2) receiving prenatal or early postnatal care in Kern County; (3) resident of Kern County for at least 6 months; (4) currently pregnant or delivered a child within the previous 12 months; (5) “high-risk”, i.e. had a history of syphilis infection, incarceration, current or previous substance use, and/or multiple sex partners; (6) have a means of being contacted; and (7) consent to study involvement. Approximately 10 women across all recruitment groups declined participation because they lacked the time or did not feel comfortable discussing sexual health in a group setting.

Participants were only interviewed one time so there was no follow up and thus, no attrition. A total of 5 focus-group-discussion (FGDs) were conducted with a total of 42 pregnant or recently pregnant persons. Pregnant and postpartum participants received a $25 gift card for their time. Prenatal care providers were recruited from the primary health systems in Kern County: Omni Family Health, Clinica Sierra Vista, and Kern Medical. Eligibility criteria for prenatal care providers included (1) current prenatal care provider in Kern County and active for ≥ 6 months; (2) ≥ 50% of their patient population is considered “high risk”; (3) have a means of being contacted; and (4) consent to study involvement. We were unable to contact many of the prenatal care participants we had hoped to recruit because they did not respond to our contact efforts, but there were no providers who refused to participate. A total of 8 in-depth-interviews (IDIs) were conducted with 8 prenatal care providers. Prenatal care providers received a $50 gift card for their time.

### Data collection

All data were collected between September 2018 and January 2019. IDIs were conducted with prenatal care providers in person or on the telephone with a trained field researcher. IDIs were asked to share their age, gender/gender identify, medical profession, and years of practice. A semi-structured question guide was used and provided to interviewees to focus the discussion on the provider’s clinical knowledge of syphilis and management during pregnancy, and their opinions of structural determinants of their patients’ health-seeking behavior. The IDIs were audio recorded and later transcribed with participant consent. Each IDI lasted approximately 60 minutes. FGDs with pregnant and postpartum participants were conducted at the location from which the participants were recruited and were moderated by one field researcher while a second assisted with logistics of the group (e.g., managing latecomers). FGD participants were asked to if they identified as Hispanic, Latino/a or of Spanish origin; FGD participants were asked to describe their race and prompted to select from all that apply from the following: White, Black/African American, American Indian/Alaska Native, Asian, Pacific Islander, Other (Specify). A semi-structured question guide was used and provided to interviewees to focus the discussion on their knowledge and perceptions of sexually transmitted infections (overall) and syphilis (more specifically), their beliefs on trends of accessing prenatal care in Kern County, and what they thought were the biggest challenges in accessing and utilizing healthcare in their community. The FGDs were audio recorded and later transcribed with participant consent. One FGD was conducted in Spanish; during the transcription process, the audio recordings were first translated into English by MN and later went through a quality checking process by a Spanish speaking research assistant at UCSD who made corrections as needed. Each FGD lasted approximately 90 minutes. No one else other than the researcher and participants were present during interviews. Interview transcripts were not returned to participants, and participants did not provide feedback on the findings. No repeat interviews were carried out. Data was collected until saturation—or the point at which the retrieval of additional perspectives or information ceased—was met.

### Qualitative thematic analysis

Interview transcripts were read, coded and analyzed by EC, CS and JW to establish inter-rater reliability and reproducibility. Reflexive thematic analysis and deductive methodology as described by Braun and Clarke was employed for qualitative data analysis to identify structural determinants of congenital syphilis vulnerability [13]. A coding system, informed by the Structural Competency Working Group and Reproductive Justice Frameworks, was developed to identify structural determinants of congenital syphilis vulnerability from interviews [14, 15]. A unique codebook was developed for analysis of IDIs with prenatal care providers and FGDs with pregnant and postpartum persons. We employed Dedoose® (SocioCultural Research Consultants, UCLA), a computer assisted qualitative data analysis software, to code interview transcripts which were then exported to Microsoft® Excel. Themes common to interviews with pregnant and postpartum persons included language barriers, transportation barriers, economic systems, judicial/carceral systems, immigration systems, gender-based sexual or domestic violence, insurance status, and health education systems. Themes common to interviews with prenatal care providers included clinical education and training in sexually transmitted infections, management of syphilis in pregnancy, and funding and availability of syphilis testing and treatment services. EC, CS, SC reviewed coded interviews separately to avoid bias; collective discussions were later held between EC, CS, SC, MPG, and JW to discuss and resolve coding differences and to assess relevance to clinical outcome. As candidate themes emerged from coded interviews, they were compared against the original dataset for internal and external validity.

### Ethics

All research materials were developed in collaboration with the Centers for Disease Control and Prevention and March of Dimes. Research materials were approved by the Centers for Disease Control and Prevention Office of Management and Budget. Research materials were reviewed and approved by the University of California, San Diego Human Research Protections Program and the Tulane University Human Research Protection Program. Written consent was obtained from all participants. A copy of the consent form and a description of the study was provided to all participants. Contact information of the principal investigator and the approving institutional review boards was provided to participants to call with questions or concerns.

## Results

A total of 50 people participated in our study: 8 prenatal care providers and 42 pregnant and postpartum persons. The 8 prenatal care providers included 3 public health nurses, 2 obstetrician/gynecologists, one nurse practitioner, one registered nurse, a health care consultant, a clinical supervisor, and a medical investigator for Kern County Public Health Services Department. Average age of providers was 52 years; average years in practice was 14. Of the 42 pregnant and postpartum participants, 9 were recruited from a local residential rehabilitation program, 10 from a non-residential rehabilitation program, 8 from a transitional living community for those with substance use disorders, 5 from a domestic violence and sexual assault organization, and 10 from the greater community in Kern County. Average age of participants was 31; 11% were pregnant at the time of the interview and 88% were within 1 year postpartum. In self-reported race/ethnicity, 50% of participants identified as being of Hispanic, Latino/a or Spanish origin; 36% as white alone (not Hispanic or Latino/a), 12% as Black/African American alone, 5% as American Indian/Alaska Native and 7% indicated two or more races. In self-reported income, 94% identified as having an annual income of less than $20,000. We identified and organized the findings to describe several structural determinants of congenital syphilis vulnerability at the patient-level and the provider-level. Structural barriers experienced by pregnant and postpartum persons to prenatal care access and utilization included (1) burdens of poverty; (2) stigma around substance use in pregnancy; (3) citizenship status; (4) lack of healthcare coverage; (5) low sexual health literacy; and (6) gender inequality. Structural barriers experienced by prenatal care providers in congenital syphilis prevention included (1) limited guidance on clinical management of syphilis in pregnancy; (2) decay in public health infrastructure; and (3) inadequate support for managing patients’ social comorbidities.

### Patient-level structural barrier: Poverty

Burdens of poverty presented major barriers to patients seeking prenatal care. Co-pays and out-of-pocket payments deterred patients from seeking medical care. One interviewee explained, “I have been living out in the streets and I got cut off from the Department of Human Services…I didn’t really go to the doctor or I would get billed” (pregnant participant, residential rehabilitation program). Many women in our study expressed that they were unable to take time off from their job for a prenatal appointment because of a lack of paid leave. Unreliable means of transportation made it difficult to get to appointments, or interfered with other employment and family demands: “You don’t make it to the appointments because it’s going to take too long on the bus or you know you won’t make it back in time or you don’t have nobody to pick up the kids at school” (pregnant participant, transitional living community). Housing insecurity meant providers could not reliably contact patients, resulting in loss-to-follow up: “She was a homeless person in her 20s…by the time the test came back, she was nowhere to be found” (prenatal care provider); “You don’t always have a phone or know they are not going to make it back in” (medical investigator).

### Patient-level structural barrier: Stigma

Stigma around drug use in pregnancy and fear of legal action led many patients to under-utilize prenatal care services. One interviewee from the transitional living community told us, “[Women] are afraid to go and seek medical attention because they are on drugs or because there might be abuse going on and they’re scared because [doctors] are mandated reporters. There are a lot of girls that don’t go because they are afraid to have their child taken from them” (postpartum participant, transitional living community). Interviews with prenatal providers revealed empathy toward this fear and a recognition of fault: “Health care providers are focused on the child primarily… If all we’re doing is running on the same treadmill of making sure the child is as safe as possible but not engaging with the mother, we’re not doing what we should be doing” (prenatal care provider).

### Patient-level structural barrier: Citizenship status

Fear of deportation and hesitancy to share immigration details with medical providers was another major deterrent in seeking prenatal care. Pregnant persons without documentation were afraid of detainment at medical offices by Immigration and Customs Enforcement officials: “For people in this country without immigration papers, it’s difficult…Due to the negative political climate, oftentimes a person prefers to keep quiet before talking with the doctor” (Spanish-speaking pregnant participant, community). Prenatal providers attempted to quell these fears, but with minimal success: “I could tell them, ‘Oh, you shouldn’t be afraid. You can come to the clinic. Nobody’s going to catch you if you don’t have documentation.’…they still don’t believe it” (prenatal care provider).

### Patient-level structural barrier: Lack of healthcare coverage

Pregnant and postpartum participants described a number of reasons for lack of healthcare coverage, including unemployment and unawareness of affordable healthcare coverage options: “When I got pregnant I didn’t [see a doctor]. I didn’t know I could get health insurance right away…I was young” (postpartum participant, domestic violence and sexual assault organization). Immigration status was another barrier to attaining healthcare coverage: “It’s much easier for someone born here to get MediCal, especially with a social security card and ID. Someone who was not born here has to jump through a lot of hoops to get MediCal” (postpartum participant, residential rehabilitation program). For many, incarceration and the correctional health system was their first presentation to primary care. A postpartum participant shared, “I’m grateful that I went to jail cause I got the medical attention that I needed. When I wasn’t in custody I applied for MediCal so I could get prenatal care, but it was very difficult” (postpartum participant, transitional living community).

### Patient-level structural barrier: Low sexual health literacy

Major gaps in knowledge of sexually transmitted infections and poor awareness of the complications in pregnancy were apparent from interviews. The following are responses from pregnant and postpartum participants at a nonresidential rehabilitation program to the question, “How do STDs (sexually transmitted diseases) start?”:

“Someone’s dirty.”

“I know it’s sex but how does the person with the sex get it?”

“I think chlamydia is like dirty sex.”

“Woah! I thought that was syphilis.”

“Gonorrhea sounds gross.”

“The clap.”

“It sounds green.”

Lack of a standard and comprehensive sexual health curriculum led many participants to seek and depend on non-scientific information from internet sources, colloquial folk-tales, peer word-of-mouth, and community crisis pregnancy centers which persuade women to choose adoption or parenting instead of an abortion [16]. Prenatal care providers expressed concerns about the reliability, credibility and validity of the information around sexual health and pregnancy their patients received. Regarding crisis pregnancy centers specifically, prenatal care providers expressed concern that the non-medically licensed facilities operating under a faith-based organization were providing inaccurate and propagandic information: “[Crisis pregnancy centers] are trying to counteract anyone getting an abortion, but they’re women’s clinics run by churches. They hurry up and give you an ultrasound and say you’ll see your baby. You will want to get rid of your baby but they don’t talk about congenital syphilis there” (prenatal care provider). Stigma around sexually transmitted infections left many patients believing they were unworthy of treatment, or feared having its documentation in their chart for insurance and legal reasons: “In all honesty, it’s what they believe in themselves and their self-worth. If they believe that they’re not worthy of treatment, they stop themselves” (prenatal care provider).

### Patient-level structural barrier: Gender inequality

Complex sexual relationships (i.e. transactional sex, gender based domestic violence, or multiple sexual partners) and gendered dynamics (giving men higher status than women) in medical settings left many pregnant persons with low decision making power to seek prenatal care. A medical investigator recalled how one patient was given strict curfews and protocols by her sex partner to follow: “I would see her frequently. The person that was controlling her would let her go to the particular place that I was at or her father’s home, and that was it. And she had an hour to be back or he’d come looking for her. And you try the best that you can. We got her linked up to some services for a while, and for whatever reason because she was pregnant with his kid, she ended up back in that situation again” (medical investigator).

### Provider-level structural barrier: Limited guidance on clinical management of syphilis in pregnancy

While the Centers for Disease Control and the American College of Obstetricians and Gynecologists have set forth clear guidelines on the management of syphilis in pregnancy, there was low awareness of these guidelines among the prenatal care providers interviewed in this study. Lack of infrastructure to disseminate knowledge of and training for existing guidelines left many prenatal care providers feeling ill-equipped to manage syphilis in pregnancy: “I never received formal training on treatment. It was merely a conversation with Public Health” (prenatal care provider). As a result, a large number of prenatal providers referred patients with syphilis to the Kern County Department of Public Health and Medical Center. Overburdened, these medical centers often could not schedule appointments “for 3 or 4 months” after the referral (medical investigator), resulting in untimely prenatal care and low patient retainment. A public health official described disempowerment of providers: “The providers will be all, ‘This is a high-risk thing. We can’t do this. We’re going to send you to Kern Medical… They’re sending women over there because they’re thinking ‘Oh my god, I can’t—I can’t do this’” (medical investigator).

### Provider-level structural barrier: Decay in public health infrastructure

A challenge characterizing the congenital syphilis epidemic included a lack of public health and preventive care infrastructure. A prenatal care provider described inadequate resource allocation to congenital syphilis prevention as “a shame, because I think it shouldn’t be conceived as an expense to get people treated. It’s an investment because these people are American citizens, born with a disability, and physically ill for the rest of their lives. I think there should be some money. They pick a couple of million dollars in this issue, and you can save BILLIONS of dollars in the future” (prenatal care provider). The high cost and burdensome storage requirements of Bicillin® was a major contributor to this resource shortage. Providers described a tendency to rely on County Health Departments to buy and store Bicillin®, rather than purchasing and storing within the clinic, for cost saving. A representative from the county Public Health Department told us, “One of the big barriers [to congenital syphilis prevention] is that we have Bicillin because we’re the Health Department. Doctors’ offices don’t often have it because it costs a lot and it has to be held at certain temps and they may not have room for it” (medical investigator).

### Provider-level structural barrier: Inadequate support for managing patients’ social comorbidities

Prenatal care providers described the challenge of managing the complex medical and social comorbidities of their patients with syphilis. Providers estimated 50–80% of their patients with syphilis were lost to follow-up: “It was very difficult to have these women be committed or follow through. I would say less than 20 percent were actually compliant” (prenatal care provider). Comorbid alcohol and substance use, housing insecurity and competing priorities secondary to poverty (e.g. food insecurity, unstable housing, unreliable transportation, inconsistent income) were described as contributors to “non-compliance” with treatment and discontinuities in prenatal care.

## Discussion

Our study detailed the structural barriers that potentially contribute to lack of prenatal care access and utilization and congenital syphilis prevention in Kern County, CA. Descriptive qualitative analysis of interviews with pregnant and postpartum persons indicate that poverty, substance use, citizenship status, lack of healthcare, low sexual health literacy and gender inequality may interfere with recommended care during pregnancy. Furthermore, analysis of interviews with prenatal care providers revealed that a decaying public health infrastructure and lack of adequate support in managing patient’s medical and social comorbidities may delay timely syphilis treatment.

Literature on the social determinants of health suggests that people experiencing social vulnerability and economic deprivation are less likely to engage in healthcare as necessary [17]. Additional support for socially vulnerable pregnant persons will be important to reduce congenital syphilis cases attributable to maternal vulnerability. Expanding enrollment in MediCal and MediCaid, eliminating co-pays, providing transportation and child care, and offering same day test-and-treat may help facilitate engagement in health care and early identification of pregnant persons or persons of reproductive age and their partners with syphilis. Creating culturally-competent prenatal programs and case management for persons with syphilis may help alleviate barriers to medical care attributable to social, cultural and linguistic differences and encourage greater participation and retention in care. Patients who have been affected by immigration and correctional violence face additional barriers to healthcare coverage due to fear of deportation, detention and bias. An analysis of the structural racism historic to medicine and actionable plans to change course will be necessary to engage with this uniquely vulnerable population. A formal declaration of medical services as a sanctuary and advocating for the removal of police presence in healthcare institutions should be further considered. Finally, developing a standard comprehensive reproductive health curriculum will be essential for the prevention of all sexually transmitted infections, including syphilis, and reducing the burden of their reproductive health consequences on socially vulnerable communities.

Our analysis highlights the role played by the public health system and prenatal care providers in contributing to congenital syphilis cases. Public health cuts have impacted clinics’ and pharmacies’ ability to carry the standard treatment for syphilis, Bicillin®. Major price differentials for clinics that do not qualify for 340B drug pricing (~$0.37/box vs $3,869.63/box manufacturer’s suggested retail price), unique storage requirements, disutility for treating other conditions, and reimbursement issues with Medi-Cal and FPACT all hinder accessibility to syphilis treatment [18]. Furthermore, lack of clinical guidance and support for prenatal care staff can make identifying and treating persons with syphilis more challenging. A recent study of prenatal care providers revealed that most providers admit little to no notification from public health departments of the need to more actively screen for syphilis in pregnant persons [7]. Reinvesting in public health departments, engaging providers in screening and surveillance guidelines, and creating wrap-around services that link prenatal care with programs that serve vulnerable women will be important to reduce congenital syphilis cases attributable to health care provider missed opportunities.

While our study provides important insight into this emerging epidemic, there are potential limitations. First, the data for this study was collected from a single site and a highly selected subset of people from Kern County and is therefore limited in its generalizability to other populations. However, the data still provides an enriched representation of the experiences of a uniquely vulnerable population and provide important insights into the drivers of the current epidemic and possible solutions. Second, eligibility criteria of pregnant and postpartum participants in the original study did not mandate a confirmed diagnosis of syphilis or congenital syphilis. Therefore, though participants were able to speak to experiences of how congenital syphilis affected close family members or their community, the results of this study do not provide direct analysis of adverse health outcomes from un- and under-treatment of syphilis and vertical transmission. Lastly, to protect patient privacy, the race and ethnicity of the participant were not made available for analysis in the de-identified transcriptions. Therefore, direct implications regarding structural racism within this study were not made.

A review of the literature highlights how little is known about the political, social and economic forces which may present barriers to prenatal care and congenital syphilis prevention. Our results suggest that there are several important health system and policy-level factors contributing to congenital syphilis vulnerability. Exploring the social determinants of congenital syphilis will further our understanding of this re-emerging epidemic and help up to identify practical policy intervention that can help control the epidemic. Qualitative studies on health disparities are necessary in order to identify ways in which to address social and societal determinants.

## Conclusion

This is a qualitative assessment for the structural barriers in prenatal care that contribute to congenital syphilis. The findings from this study provide a richer understanding of how structural inequalities give rise to disparate health outcomes in the setting of congenital syphilis. Despite increasing attention being paid to the structural determinants of health as a root cause of inequality, interventions continue to prioritize individual psychosocial and behavioral risk identification as solutions to alleviating disparities; however, these models fail to adequately address the political, social and economic context in which these individual factors arise. We conclude that an appropriate congenital syphilis response will require:

1. alleviating the burdens of poverty that make it more difficult for pregnant patients to access and utilize prenatal care services, such as through eliminating co-pays, providing transportation assistance to prenatal care sites, streamlining same day diagnosis and treatment, and co-location of prenatal care with existing health and social services;
2. an examination of implicit bias within prenatal care and implementation of a reproductive justice agenda aimed to address the relationship between disparities in reproductive health and the unequal treatment of persons who use illicit substances, such as through implementing implicit bias training and Equity and Inclusion leadership;
3. ensuring visibility, accessibility and continuity of public health insurance, such as through a universal health care;
4. developing and implementing a standard sexual health education curriculum formulated by educated health professionals in school systems and ensuring availability of family planning services;
5. engaging with health care providers to expand screening guidance, surveillance and coordinate provider education on congenital syphilis as a priority health issue;
6. empowering states to work with providers serving uninsured and underinsured patients to expand access to Bicillin® at 340B drug pricing, and increase monthly orders of Bicillin® by the Public Health Pharmacy to be distributed across STD clinics.

Greater attention must be given to the issues that disproportionately affect vulnerable populations. These findings highlight several structural barriers that potentially contribute to lack of prenatal care access and utilization and congenital syphilis prevention in Kern County, CA. The response to the congenital syphilis epidemic will require an examination of the complex context of social determinants of health in which persons diagnosed with syphilis live in, and a discussion of the ways in which we can optimize treatment and prevention of syphilis infections.

## Supporting information

S1 Checklist COREQ (COnsolidated criteria for REporting Qualitative research) checklist. (PDF)
